# FILTUS: a desktop GUI for fast and efficient detection of disease-causing variants, including a novel autozygosity detector

**DOI:** 10.1093/bioinformatics/btw046

**Published:** 2016-01-27

**Authors:** Magnus D. Vigeland, Kristina S. Gjøtterud, Kaja K. Selmer

**Affiliations:** ^1^Department of Medical Genetics, Oslo University Hospital and University of Oslo, Oslo N-0424,; ^2^Department of Research, Cancer Registry of Norway, Oslo N-0304, Norway

## Abstract

**Summary:** FILTUS is a stand-alone tool for working with annotated variant files, e.g. when searching for variants causing Mendelian disease. Very flexible in terms of input file formats, FILTUS offers efficient filtering and a range of downstream utilities, including statistical analysis of gene sharing patterns, detection of *de novo* mutations in trios, quality control plots and autozygosity mapping. The autozygosity mapping is based on a hidden Markov model and enables accurate detection of autozygous regions directly from exome-scale variant files.

**Availability and implementation:** FILTUS is written in Python and runs on Windows, Mac and Linux. Binaries and source code are freely available at http://folk.uio.no/magnusv/filtus.html and on GitHub: https://github.com/magnusdv/filtus. Automatic installation is available via PyPI (e.g. pip install filtus).

**Contact:** magnusdv@medisin.uio.no

**Supplementary information:** Supplementary data are available at *Bioinformatics* online.

## 1 Introduction

Recent years have seen a revolution in Mendelian disease gene identification thanks to high-throughput sequencing (HTS) methods, in particular whole-exome sequencing (WES). Most of the released software for downstream analysis is aimed at bioinformatically trained users, thus posing a challenge for many medical researchers wanting to do hands-on analysis of HTS data. To accommodate this, we introduce a program (FILTUS) offering advanced tools for identifying disease-causing variants in an easy-to-use graphical environment.

Several programs for manipulating variant files have been published, including VarSifter (Teer *et al.*, 2012) and the web-based EVA (Coutant *et al.*, 2012). In addition to efficient browsing and filtering, FILTUS offers several specialized analysis tools aimed at Mendelian disease projects. These include statistical evaluation of variant sharing among patients, *de novo* detection in trios and autozygosity mapping.

FILTUS accepts virtually any variant files, in contrast to most existing programs which are limited to Variant Call Format (VCF) or other specific input formats. Typical examples of non-standard formats are VCF files with additional columns, and files produced by Annovar (Wang *et al.*, 2010). Although FILTUS is primarily intended for WES-scale data, whole-genome data can be analyzed by using the built-in prefiltering functionality.

Some countries have strict regulations requiring offline handling of all human sequencing data, thus making it impossible to use web-based tools or to download information during analysis. FILTUS is ideal for such working conditions, requiring no installation, being completely self-contained and offline.

In summary, the main features of FILTUS include:

- Stand-alone, offline desktop tool with user-friendly GUI
- Very flexible in terms of input file formats
- Simultaneous analysis of up to several hundred exomes
- Fast, versatile filtering, including summary table
- Column summaries and quality control (QC) plots
- Export to MERLIN-format (for linkage analysis)
- Creating and manipulating in-house variant databases
- Statistical gene prioritizing, detection of *de novo* mutations in trios and autozygosity mapping

## 2 Methods and results

### 2.1 Statistical evaluation of gene sharing

A common strategy for Mendelian disease gene identification is to compare WES data from unrelated patients with the same phenotype. The basic idea is to apply strict filters, leaving only potentially disease-causing variants compatible with the inheritance model, and then look for genes where these variants are enriched among the patients. In many cases this method produces a long list of genes with no obvious ranking. FILTUS implements a statistical model (Zhi and Chen, 2012) for evaluating the significance of each gene in the output (Supplementary Material S1).

### 2.2 Detection of *de novo* mutations

There is an emerging understanding that *de novo* mutations are a major cause of Mendelian disorders. As a result, trio sequencing has become a popular design when faced with an isolated patient with healthy parents (Chong *et al.*, 2015). To overcome the practical challenge of false positives and negatives, Bayesian methods are typically used, as in DeNovoGear (Ramu *et al.*, 2013). FILTUS implements a similar approach, computing posterior *de novo* probabilities from the genotype likelihoods provided by the variant caller (Supplementary Material S2). We compared FILTUS with DeNovoGear by applying both to a publically available trio data set. The results were very similar, particularly when using filters typical in clinical settings (Supplementary Material S3). In addition to posterior probabilities, FILTUS computes ALT allele percentages for each trio member, facilitating ranking and filtering.

### 2.3 Autozygosity mapping: the AutEx algorithm

Autozygosity (or homozygosity) mapping (Lander and Botstein, 1987) is a powerful method for mapping recessive disorders. Traditional sliding-window approaches as offered by PLINK (Purcell *et al.*, 2007) are designed for dense, evenly distributed SNPs and are not optimal for exome data. Better methods have recently been proposed, e.g. H3M2 (Magi *et al.*, 2014), and the -roh command of BCFtools (Li *et al.*, 2009), but these require skillful bioinformatic handling of sequence data.

As an alternative, we introduce the AutEx algorithm for detecting autozygous regions directly from variant files. Our approach is based on a hidden Markov model (Leutenegger *et al.*, 2003) described in Supplementary Material S4. The user specifies an approximate parental relationship and a column with allele frequencies (if available). The output provides details of each estimated autozygous segment, and can be directly used for filtering. Zoomable plots show the detected regions with surrounding variants. An example of a disease gene identification aided by AutEx is given in Supplementary Material S5.

We compared AutEx with traditional homozygosity mapping to validate its performance, using data from a child of first cousin parents for which both dense SNP genotypes and WES data were available. Taking the SNP-based homozygous segments (as detected by PLINK) as the true segments, AutEx applied to the WES variants exceeded 95% for both true positive and true negative rates (Supplementary Material S6).

### 2.4 Visualizations

FILTUS offers various plots to aid QC of the variant files: gender estimation (based on X-chromosomal heterozygosity levels), private variants (compared with the other samples) and autosomal heterozygosity level (examples given in Supplementary Material S7). In addition histograms and scatter plots can be made from any numerical columns.

## 3 Discussion

FILTUS has been used in many successful disease gene identifications, some of which are published (Baroy *et al.*, 2015; Fjaer *et al.*, 2015; Hansen *et al.*, 2015; Pedurupillay *et al.*, 2015) and others currently in preparation. FILTUS runs on Windows, Mac and Linux (see Supplementary Material S8 for supported versions) and is actively maintained and developed. We believe it to be a valuable contribution to the computer toolset of researchers and clinicians working with HTS variant data, especially those without access to specialized bioinformatics resources.

## Supplementary Material

Supplementary Data
