# Supplementary material for: FILTUS: a desktop GUI for fast and efficient detection of disease-causing variants, including a novel autozygosity detector
Source: Bioinformatics. 2016 Jan 27;32(10):1592–4. doi: 10.1093/bioinformatics/btw046 (PMC4866527; doi:10.1093/bioinformatics/btw046)
Supplement: Supplementary Data [file supp_32_10_1592__index.html]

FILTUS: a desktop GUI for fast and efficient detection of disease-causing variants, including a novel autozygosity detector — FILTUS: a desktop GUI for fast and efficient detection of disease-causing variants, including a novel autozygosity detector — Supplementary Data 

# FILTUS: a desktop GUI for fast and efficient detection of disease-causing variants, including a novel autozygosity detector

## Supplementary Data

files

- Supplementary Data - pdf file
