# Supplementary material for: FILTUS: a desktop GUI for fast and efficient detection of disease-causing variants, including a novel autozygosity detector
Source: Bioinformatics. 2016 Jan 27;32(10):1592–4. doi: 10.1093/bioinformatics/btw046 (PMC4866527; doi:10.1093/bioinformatics/btw046)
Supplement: Supplementary Data [file supp_btw046_filtus_supplementary.pdf]

FILTUS: a desktop GUI for fast and efficient detection of disease-causing variants, including a novel autozygosity detector

Magnus D. Vigeland, Kristina S. Gjøtterud and Kaja K. Selmer

Supplementary material and examples

S1 Statistical gene sharing analysis

FILTUS implements the statistical model described in (Zhi and Chen, 2012) for ranking the genes after a gene sharing analysis of unrelated patients. The model rests on a basic assumption that variants surviving strict filtering are uniformly distributed along the genome. One can then show that for a given gene, the number of patients with at least 1 variant in the gene (or at least 2 variants, for recessive disease models) is approximately binomially distributed. The distribution depends on the gene lengths, genetic model, and the total number of variants after filtering. By comparing the observed gene sharing counts to these theoretical distributions, one can assign a P-value to each gene. Given the simplistic assumptions made on the distribution of variants, these P-values should not be trusted blindly. However, for ranking and gene prioritization they have been proven to work well (Zhi and Chen, 2012).

Note: The gene sharing analysis requires the variants to be annotated with gene association. This step is upstream of FILTUS and can be performed by a variety of variant annotation tools, for instance VEP (McLaren et al., 2010) or Annovar (Wang et al., 2010)

S2 Detection of *de novo* mutations

FILTUS offers a fast and user friendly functionality for detection of *de novo* mutations (DNM). This analysis requires that the input file contains variants for a child and both parents, and that is uses VCF format for the genotype data. In particular, the format column must include fields GT (genotype), AD (allele depth) and PL (phred scaled genotype likelihoods). Apart from the genotype columns, the file itself does not have to be in VCF format.

DNM detection is performed in two stages:

1. The GT field is used to identify variants with a *de novo* genotype pattern
2. For each identified variant, FILTUS computes the following statistics for ranking and filtering:
    i. Posterior *de novo* probabilities, (using PL field and population allele frequencies)
    ii. For each trio member: Observed percentage of reads with the ALT allele (using AD field)

The computation of posterior probabilities is described in detail further down. The ALT percentages can be useful for reducing the number of false positives, for instance by setting hard thresholds like child > 35 % and parents < 5 %.

Recognized de novo *genotype patterns*

FILTUS recognizes the following *de novo* genotype patterns (father + mother = child):

- Autosomal:
  - 0/0 + 0/0 = 0/1
  - 0/0 + 0/0 = 1/1
  - 0/0 + 0/1 = 1/1
  - 0/1 + 0/0 = 1/1
- X-linked, child is boy:
  - 0 + 0/0 = 1
- X-linked, child is girl:
  - 0 + 0/0 = 0/1
  - 0 + 0/0 = 1/1
  - 0 + 0/1 = 1/1

A variant is treated as X-linked in this context only if it is located outside of the pseudoautosomal regions *PAR1* and *PAR2* on the X chromosome. Multiallelic generalizations of the above patterns are also caught. However, combinations with any of the following properties are treated as benign and discarded from further analyses:

- The *de novo* allele is 0 (= REF). Example: 1/1 + 1/1 = 0/1.
- Child genotype equals either of the parents. Example: 0/0 + 1/1 = 1/1.
- Missing genotype in any trio member.
- A male trio member is reported as heterozygous for an X-linked variant.

Posterior de novo *probabilities*

In the remainder of this section we describe the Bayesian computation of posterior *de novo* probabilities. For a fixed position, let $G = (g_m, g_f, g_c)$ be any combination of genotypes for the mother, father and child, respectively. The posterior probability of G is then, by Bayes' rule:

$$P(G \mid data) \propto P(G) \cdot P(data \mid G) \qquad (*)$$

Assuming the parental genotypes to be independent, the prior *P(G)* is computed as

$$P(G) = P(g_m) \cdot P(g_f) \cdot P(g_c | g_m, g_f), \qquad (**)$$

where $P(g_m)$ and $P(g_f)$ are the population frequencies of the parental genotypes. These are approximated from the allele frequencies, assuming Hardy-Weinberg equilibrium. The final term of (**) is very important: Rather than using pure Mendelian transmission probabilities (which would give zero for a true *de novo*, e.g. with $g_m = g_f = 0/0$ and $g_c = 0/1$) we condition on *de novo* status in each parental meiosis. The prior *de novo* mutation rate defaults to 1e-8 but can be modified by the user. In particular, it should be noted that the same prior is used for point mutations and indels (unlike e.g. DeNovoGear). Although biologically slightly inaccurate, our experience is that this simplification has little effect on the output with high quality data (see also section S3).

Back to (*), we factor the likelihood of G as $P(data \mid G) = GL(g_m) \cdot GL(g_f) \cdot GL(g_c)$, where $GL(g_m) = P(data_m \mid g_m)$ is the likelihood of genotype g_m for the mother, and similarly for the father and child. The GL values are computed by most variant callers, but are typically reported in its phred-scaled version PL. This scaling amounts to $GL(g) \propto 10^{-PL(g)/10}$. Putting it all together, the complete posterior distribution of G is determined by

$$P(G \mid data) \propto P(g_m) \cdot P(g_f) \cdot P(g_c | g_m, g_f) \cdot 10^{-[PL(g_m)+PL(g_f)+PL(g_c)]/10}$$

The posterior *de novo* probability reported by FILTUS is $P(G_{dn} \mid data)$ where G_{dn} is the identified *de novo* genotype combination.

S3 Comparison with DeNovoGear

To validate and assess the performance of the DNM detection algorithm of FILTUS, we compared it with DeNovoGear (Ramu et al., 2013).

As test case we used benchmark data from the Genome In a Bottle Consortium (GIAB) (Zook et al., 2015), specifically the exome sequencing data of the Ashkenazim Jewish trio. The VCF file resulting from joint variant calling of this trio is available from the GIAB ftp site (ftp://ftp-trace.ncbi.nlm.nih.gov/giab/ftp/data/AshkenazimTrio/analysis/ OsloUniversityHospital_Exome_GATK_jointVC_11242015/HG002-HG003-HG004.jointVC.filter.vcf). We annotated the variants using Annovar (Wang, et al., 2010), and applied a filter removing low quality variants (PASS, DP>9, repeatMasker=NA, genomicSuperDups=NA). The remaining variants were then subjected to DNM detection by DeNovoGear and FILTUS, both run with default parameters. We recorded the number of variants detected by either or both programs.

The results are summarized by the Venn diagram in Figure 1A. The 5 variants detected only by DNG all had the property that the *de novo* allele was REF, which are purposely disregarded by FILTUS. Among the 66 variants reported only by FILTUS, most are easily recognizable as false positives, judging from the posterior probabilities and the ALT read percentages. For instance, 50 of these 66 had posterior probability < 0.001.

Finally, we re-did the comparison focusing on the most interesting variants in a typical realistic setting. In addition to the quality filters above, we only kept only rare (ExAC < 0.01) exonic and splice site variants. Furthermore, after running each program we disregarded variants with a reported posterior *de novo* probability less than 10%. The results are shown Figure 1B. The two programs agree almost completely in this setting, only one variant is reported by FILTUS alone. A closer inspection of this variant, a 58 bp insertion in the *KRT77* gene, suggested that it is a false positive.

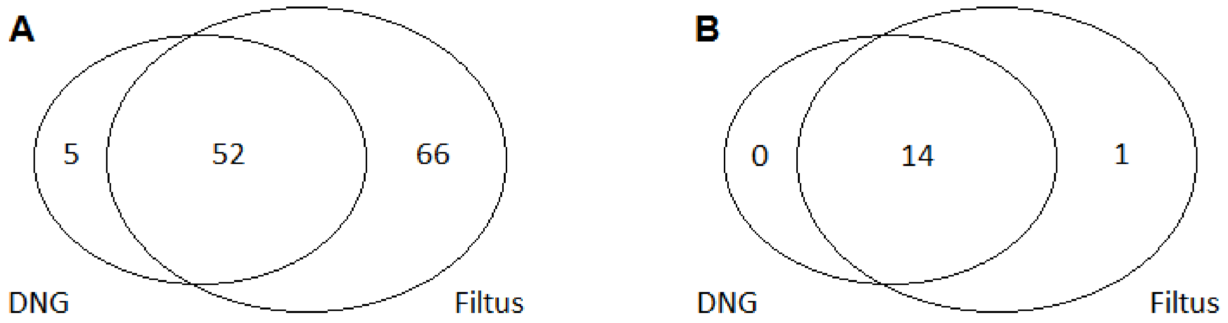

Figure 1: Venn diagrams showing the number of *de novo* variants reported by FILTUS and DeNovoGear (DNG) after liberal (A) and strict (B) filters.

S4 Autozygosity mapping: the AutEx algorithm

The goal of autozygosity mapping (or homozygosity mapping) is to recognize homozygous genomic stretches as autozygous, i.e. originating from a single ancestral haplotype. This is traditionally done by sliding-window methods, e.g. as implemented in PLINK (Purcell et al., 2007). Such approaches are intended for dense SNP genotypes and are not well-suited for exome data. We have implemented in FILTUS an algorithm (AutEx) for detecting autozygous regions directly from HTS variant files. It is based on the hidden Markov model

introduced in (Leutenegger et al., 2003), and we refer to their paper for mathematical details. In brief, the IBD process along an inbred chromosome is approximated as a Markov model with two states, IBD and not-IBD, with transition probabilities depending on the genetic map distances and also the relationship between the parents. The observed variables are the variant genotype calls, whose emission probabilities depend on allele frequencies and genotyping error rates. A standard forward-backward algorithm is applied to compute the posterior IBD probability at each variant locus. Regions where these probabilities exceed a given threshold are reported as autozygous.

The implementation uses the Decode recombination map (Kong et al., 2010) to compute genetic distances. The user must specify an approximate relationship between the parents, and indicate a column with allele frequencies, if available. The output contains the location and size (in megabases, centiMorgan and number of variants) of each autozygous segment, and can be directly used as a filter in further analyses. Each segment is described in two ways: strictly and extended. In the latter case the segment is extended to the nearest outside variant on each side. This is what one would normally use for filtering. However they can be misleading in size, e.g. if containing a whole centromere.

S5 Example: Detecting cryptic autozygosity with FILTUS

Exome sequencing was performed in two brothers with hereditary ataxia and clinical features overlapping those of Charcot-Marie-Tooth. Even though the brothers' parents were claimed to be unrelated, the AutEx algorithm was run on each brother as a routine check, using parameters consistent with a low inbreeding level. This revealed that each brother had a credible autozygous region on chromosome 5, as shown in Figures 2A and 2B. Both regions measured roughly 3.5 Mb, and overlapped each other by 2.8 Mb. Restricting to this short segment, a FILTUS search for rare nonsynonymous variants gave a single hit: A homozygous stopgain SNV in *SH3TC2*, p.Arg954Ter. This is a known pathogenic variant, causing Charcot-Marie-Tooth disease, type 4C. This was compatible with the phenotype of the brothers, and the homozygous variant was reported back to the clinical department as disease-causing.

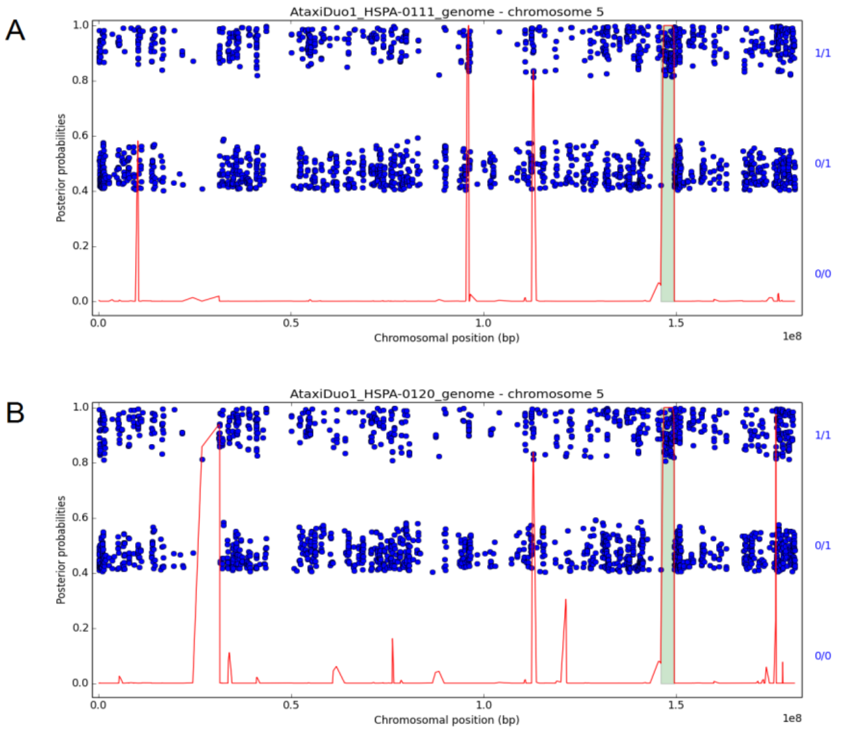

Figure 2: Autozygosity plots produced by FILTUS, showing variants on chromosome 5 from two brothers.

S6 Validation of the AutEx algorithm

To compare AutEx with traditional homozygosity mapping we used data from a child of first cousin parents. The child was genotyped with ~700k SNPs (Illumina HumanOmniExpress-24 chip) and also whole-exome sequenced to > 50x coverage. From the SNP genotypes we obtained a set of homozygous regions by using the --homozyg functionality of PLINK (with default parameters). We then aimed to replicate these findings using only the exome data. After removing variants of poor quality we applied AutEx, specifying a first cousin parental relationship.

The results are shown in Table 1. The SNP-based homozygosity mapping using PLINK identified 29 homozygous regions larger than 1.5 Mb, with total length 244 Mb. In comparison, the AutEx algorithm of FILTUS yielded 49 regions with total length 340 Mb. Both the true positive and true negative ratios were above 95% when comparing to the SNP-based results.

Data	Method	#Regions	Mb	True pos	True neg
**700k SNP**	**PLINK[1]**	29	244	-	-
**Ex Seq**	**AutEx**	49	340	95 %	96 %

Table 1: Comparing AutEx with SNP-based PLINK analysis.

S7 Quality control plots

The plotting functionality of FILTUS includes three quality plot types, described below. We recommend filtering out variants of low quality before making these plots.

- *Gender estimation*: This shows the heterozygosity ratio of the observed X-chromosomal variants of each sample, outside the pseudoautosomal regions PAR1 (chrX:60001-2699520) and PAR2 (chrX:154931044-155260560). For males one expects values close to zero, while females typically have a heterozygosity level of around 60%. Far outliers relative to these expected values might indicate abnormalities (biological or technical).
- *Private variants*: This plot shows the number of private variants of each sample, relative to all the other loaded samples. (A variant is private for sample X relative to a given set of samples if X has the variant (in heterozygous or homozygous state) while all the other samples are homozygous for the reference allele in this position.)
- *Heterozygosity*: This shows the autosomal heterozygosity level of each sample, plotted against the number of autosomal variants.

Example 1: Trio identification. The "Gender estimation" and "Private variants" plots in combination provide a quick and easy way of checking who's who in a trio. An example is shown in Figure 3, which was made from whole-exome sequencing (WES) variants of a child and both parents, after first applying a noise-reducing filter (PASS, DP>9, GQ>39, RepeatMasker=NA, genomicSuperDups=NA). In the "Private variants" plot we see that Sample1 has close to zero private variants relative to the two others, which is what we would expect from the child. From the gender estimates it then follows that Sample1 is a girl, Sample2 the mother and Sample3 the father.

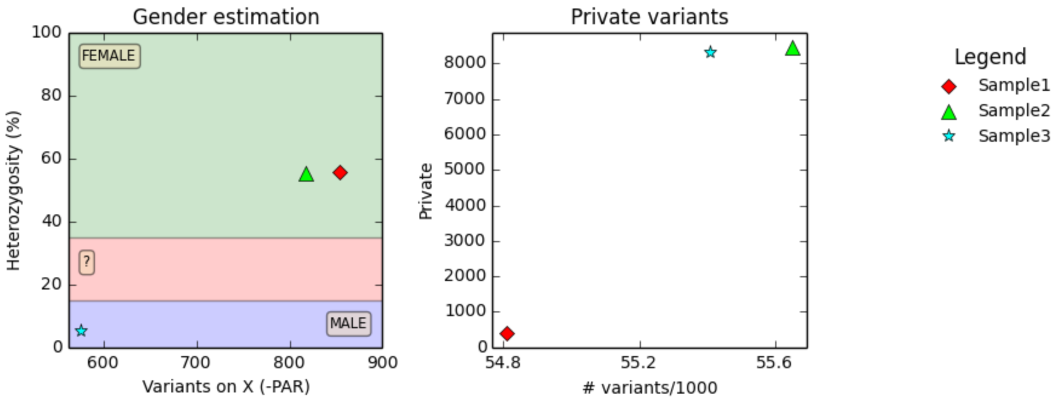

Figure 3: FILTUS plots of a trio, identifying Sample1, Sample2, Sample3 as girl, mother and father respectively.

Example 2: QC on batch of exomes. The QC plots are useful for identifying dubious or peculiar samples in a large batch. To illustrate this we loaded 100 WES variant files (exonic variants only) which should ideally form a homogeneous set: The samples were unrelated Caucasians, sequenced on the same platform and with identical alignment/variant calling pipelines.

After applying a simple PASS filter, leaving on average ~18 500 variants per sample, we made the three QC plots. The result is shown in Figure 4. Note that there is no legend in the plot this time (only with up to 12 samples), but an accompanying text file with point coordinates makes it easy to identify individual points. The plots are zoomable.

In the *Gender estimation* plot we see the males and females clustering nicely, except one clear outlier with ~30 % heterozygosity on X. Whether this is caused by biological (chromosomal) abnormalities or sequencing problems would need additional investigation of this sample. The *Private variants* plot also has a far outlier, with several times more private variants than the others. This could indicate errors of some kind – or alternatively that the sample is from a different population than the others. Finally, the *Heterozygosity* plot shows three samples with much higher autosomal heterozygosity (almost 80%) than the others. These samples also have many more variants that the others (~22 000). It is hard to imagine biological explanations for these observations, i.e. we suspect that these are technical artefacts of some kind. Further investigation of the three samples, or simply exclusion from further analysis, would be advisable.

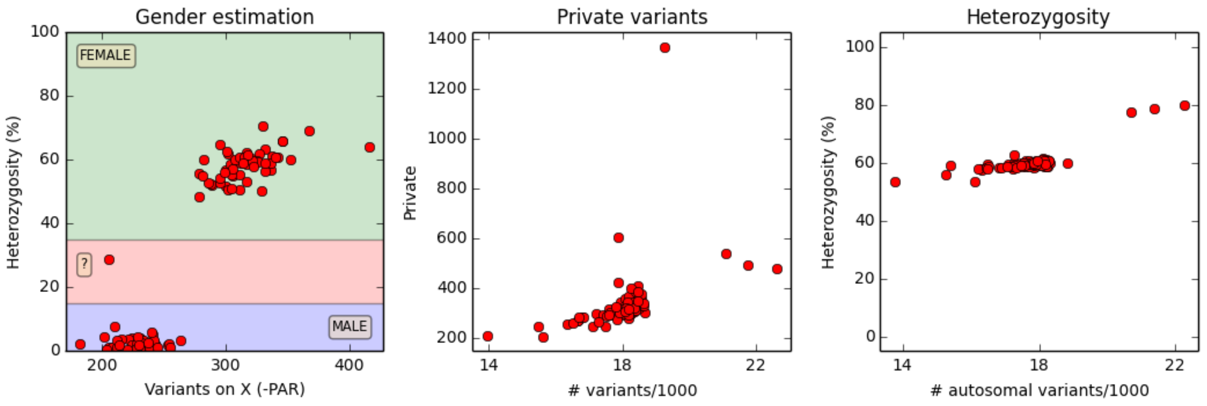

Figure 4: QC plots of 100 WES variant files.

S8 Supported operating systems

FILTUS is extensively tested with clean results on the operating systems listed in Table 2.

System	Edition
**Windows**	**Windows 7 Enterprise x64**
	**Windows 8.1 x64**
	**Windows 10 Home x64**
**Mac**	**OS X El Capitan**
**Linux**	**Ubuntu 14.04, 64-bit**
	**Red Hat Enterprise Linux 6, 64-bit**

Table 2: Tested operating systems

Acknowledgements

We thank Chantal Tallaksen, Jeanette Koht and Siri L. Rydning for clinical data regarding the ataxia patients described in section S5. Furthermore, we thank Ying Sheng for annotation of the GIAB trio and running DeNovoGear.

References

Kong, A. et al. (2010) Fine-scale recombination rate differences between sexes, populations and individuals. *Nature*, 467, 1099-1103.

Leutenegger, A.L. et al. (2003) Estimation of the inbreeding coefficient through use of genomic data. *Am J Hum Genet*, 73, 516-523.

McLaren, W. et al. (2010) Deriving the consequences of genomic variants with the Ensembl API and SNP Effect Predictor. *Bioinformatics*, 26, 2069-2070.

Purcell, S. et al. (2007) PLINK: a tool set for whole-genome association and population-based linkage analyses. *Am J Hum Genet*, 81, 559-575.

Ramu, A. et al. (2013) DeNovoGear: de novo indel and point mutation discovery and phasing. *Nat Methods*, 10, 985-987.

Wang, K. et al. (2010) ANNOVAR: functional annotation of genetic variants from high-throughput sequencing data. *Nucleic Acids Res*, 38, e164.

Zhi, D. and Chen, R. (2012) Statistical guidance for experimental design and data analysis of mutation detection in rare monogenic mendelian diseases by exome sequencing. *PLoS One*, 7, e31358.

Zook, J.M. et al. (2015) Extensive sequencing of seven human genomes to characterize benchmark reference materials. *bioRxiv*,
